# Body Size Correlates with Fertilization Success but not Gonad Size in Grass Goby Territorial Males

**DOI:** 10.1371/journal.pone.0046711

**Published:** 2012-10-04

**Authors:** Jose Martin Pujolar, Lisa Locatello, Lorenzo Zane, Carlotta Mazzoldi

**Affiliations:** Department of Biology, University of Padova, Padova, Italy; University of Milan, Italy

## Abstract

In fish species with alternative male mating tactics, sperm competition typically occurs when small males that are unsuccessful in direct contests steal fertilization opportunities from large dominant males. In the grass goby *Zosterisessor ophiocephalus*, large territorial males defend and court females from nest sites, while small sneaker males obtain matings by sneaking into nests. Parentage assignment of 688 eggs from 8 different nests sampled in the 2003–2004 breeding season revealed a high level of sperm competition. Fertilization success of territorial males was very high but in all nests sneakers also contributed to the progeny. In territorial males, fertilization success correlated positively with male body size. Gonadal investment was explored in a sample of 126 grass gobies collected during the period 1995–1996 in the same area (61 territorial males and 65 sneakers). Correlation between body weight and testis weight was positive and significant for sneaker males, while correlation was virtually equal to zero in territorial males. That body size in territorial males is correlated with fertilization success but not gonad size suggests that males allocate much more energy into growth and relatively little into sperm production once the needed size to become territorial is attained. The increased paternity of larger territorial males might be due to a more effective defense of the nest in comparison with smaller territorial males.

## Introduction

Alternative male mating strategies are hypothesized to be maintained as an evolutionary stable state, in which different behaviours allow each male type to successfully contribute to reproduction [1]. In many fish species, large dominant males defend and court females from nest sites in order to gain fertilization, often involving some sort of aggressive mate guarding. In contrast, small males that are unsuccessful in direct contests between males obtain matings using alternative tactics such as sneaking, in which males sneak into nests, release sperm, and quickly exit [1], [2]. Territorial males experience on average a lower level of sperm competition than sneaker males, as the latter always spawn when at least another male is present. Theory predicts that the higher levels of sperm competition that sneakers experience favor greater investment in gonads and greater sperm expenditure [3]. Fish with alternative mating tactics whose sperm allocation has been investigated all conform to this pattern (e.g., [2], [4], [5]). Territorial male resource allocation strategies may not only depend on the fertilization success of his sperm relative to those of his competitors, but also on the efficiency of male guarding in reducing the level of sperm competition. This efficiency is expected to increase with the relative size of the male because body size mainly determines the winner of aggressive interactions [6]–[9].

Many studies have quantified the fertilization success of males adopting each mating tactic, with territorial males usually outcompeting sneakers but with some degree of cuckoldry observed in most fish species (reviewed in [10]). However, the pattern of sperm allocation along with body size within tactic, i.e. territorial males, has rarely been studied. To our knowledge, an increased fertilization success of larger territorial males in comparison with smaller territorial males has only been reported in the three-spined stickleback [11] and in the case of dominant migrant males in salmonids [12], but their pattern of gonadal investment was not investigated.

We investigated this topic in the grass goby (*Zosterisessor ophiocephalus*), a large coastal gobiid inhabiting seagrass meadows in shallow brackish water environments of all the Mediterranean and eastern Atlantic up to the Canary Islands. The reproductive behaviour of this species follows the basic pattern observed in gobies with external fertilization by deposition of demersal eggs on a nest-sheltered substrate [13]. The grass goby presents a polygamous mating system with alternative male mating tactics, in which large territorial males (12.5–25 cm total length) build and defend a multi-chambered nest providing parental brood care, while smaller sneaker males (<12 cm total length) attempt to sneak in and release their sperm into the nest [13]–[15]. Mating tactic is ontogenetic and males behave as sneakers when small and young, and become territorial when larger and older, but continue to grow after tactic change [15]. The observations of Mazzoldi et al. [15] suggest a very intense sperm competition in the grass goby, after reporting the presence of sneakers in 70% of nests with eggs and up to six sneakers per spawning event.

The aim of our study was to analyze the relationship between body size and fertilization success within territorial males and explore gonadal investment of territorial and sneaker males in a natural population of grass goby from the lagoon of Venice (Italy; Table 1). While keeping the total number of eggs analysed high (about 700 eggs), our experimental design consisted in analyzing a high number of individuals per nest (80–90) rather than a high number of nests. This choice was justified by the low fertilization success of sneakers reported for other gobies and related species [16], [17], which could go undetected when analyzing a lower number of eggs. This way, our experimental design allowed us to accurately estimate sneaking and, in consequence, the fertilization success of territorial males.

**Table 1:** Sampling details.

Nest	Sampling date	N eggs	Territorial		NSneakers	NFemales
			TL	W		
1	05/05/2003	90	13.70	22.93	1	1
2	05/05/2003	82	14.70	28.07	1	0
3	05/05/2003	90	14.50	22.58	1	0
4	05/05/2003	82	13.80	23.52	0	1
5	01/04/2004	90	17.20	47.07	0	0
6	15/04/2004	82	19.70	69.55	0	2
7	08/06/2004	88	15.00	28.40	1	0
8	08/06/2004	84	12.50	17.12	0	2

The table includes sampling date, number of eggs per nest, total length (TL, cm) and weight (W, g) of territorial male and number of sneakers and females present at the moment of sampling.

## Results

A preliminary analysis showed that the four microsatellite loci used for parentage analysis were highly polymorphic. Number of alleles ranged from 11 to 34 across loci, while expected heterozygosities ranged from 0.42 to 0.94 (Table 2). The combined expected exclusion probabilities for all loci were extremely high, 0.91 (neither parent known) and 0.98 (one parent known), suggesting that the microsatellite loci in our study have enough power and are adequate for parentage analysis.

**Table 2:** Summary of genetic diversity at four microsatellite loci selected for parentage analysis in *Z. ophiocephalus*.

Loci	N alleles	Allele range	Frequency	H_o_	H_e_	Total exclusion probability, neither parent known	Total exclusion probability, one parent known
Zo++44m13	11	192–216	0.532	0.681	0.634	0.20	0.36
Zo++26m13	11	113–143	0.342	0.784	0.786	0.42	0.60
Zo++37m13	12	186–228	0.744	0.430	0.424	0.12	0.27
Zo++45	34	198–360	0.141	0.913	0.940	0.79	0.88
**Total**						**0.91**	**0.98**

The table includes number of alleles, allele range, frequency of the most common allele and observed (H_o_) and expected (H_e_) heterozygosities. Exclusion probabilities associated with each locus and for all loci were calculated with GERUD.

Parentage analysis was conducted for a total of 688 eggs from 8 nests (Table 3). Fertilization success of territorial males was very high, ranging from 59.2 to 92.6%, with a mean of 76.4%. The highest fertilization success of territorial males was found in the two individuals with the largest body size (nests 5 and 6; Table 1), fathering 88.9 and 92.6% of the progeny, respectively. By comparison, the fertilization success of sneakers present when the nest was collected was much lower, ranging from 4.7 to 12.9%. In all nests, a proportion of the progeny ranging from 7.4 to 40.8% was attributed to unsampled males. Female contribution was highly variable, ranging from 2.5 to 38.2%. Number of mothers ranged from 2 to 8 or more. Finally, the probability of error associated to the assessment of parentage performed using a Monte Carlo simulation ranged from 1.5 to 15.9%. In all cases, the percentage of matches of territorial males exceeded the percentage of random matches. Percentage of matches of all sneakers except one was higher than the percentage of random matches. Percentage of matches of all females exceeded the percentage of random matches, except for the females with the lowest contribution to progeny (2.5 and 2.6%, respectively).

**Table 3:** Results from parentage analysis.

Nest	% Sired				% random matches	Min N mothers
	Territorial male	Sneaker	Unsampled males	Females		
1	68.2	12.9	18.9	3.5	1.5	>8
2	76.5	7.4	16.1	–	1.8	>8
3	75.9	13.3	10.8	–	10.7	6
4	75.6	–	24.4	23.1	5.3	>8
5	88.9	–	11.1	–	15.9	2
6	92.6	–	7.4	Female A 22.2 Female B 2.5	13.0	>8
7	74.4	4.7	20.9	–	4.9	>8
8	59.2	–	40.8	Female A 38.2 Female B 2.6	7.7	5

The table includes percentage of progeny sired by the territorial male, sneaker, unsampled males and females, probability of error associated with the assessment of parentage (% random matches) and estimated minimum number of mothers (Min N mothers).

A logistic regression model of fertilization success of territorial males showed a significant advantage of larger territorial males in fertilization success (body weight: b = 0.037; S.E. = 0.007; Wald chi-square = 5.09; p = 0.007) but no effect of number of sneakers or females (Table 4). Results did not change when non-significant explanatory variables were removed from the model (body weight: b = 0.041; S.E. = 0.012; Wald chi-square = 3.34; p<0.001). The same results were obtained when extremes (territorial males from nest 6 and 8) were excluded from the analysis (p = 0.004 and p = 0.003, respectively).

**Table 4:** Logistic regression model of fertilization success.

Initial Model	*b*	SE	*t*	p
N Females	−0.246	0.243	−1.72	0.160
N Sneakers	−0.392	0.234	−1.25	0.281
Weight	0.037	0.007	5.09	0.007
**Final Model**				
Weight	0.041	0.012	3.34	<0.001

Reported is the logistic regression model of fertilization success of territorial males (proportion of progeny sired by the territorial male) in relation to body weight, number of sneakers and number of females present in the nest at the moment of sampling. SE = standard error.

**Figure 1:**
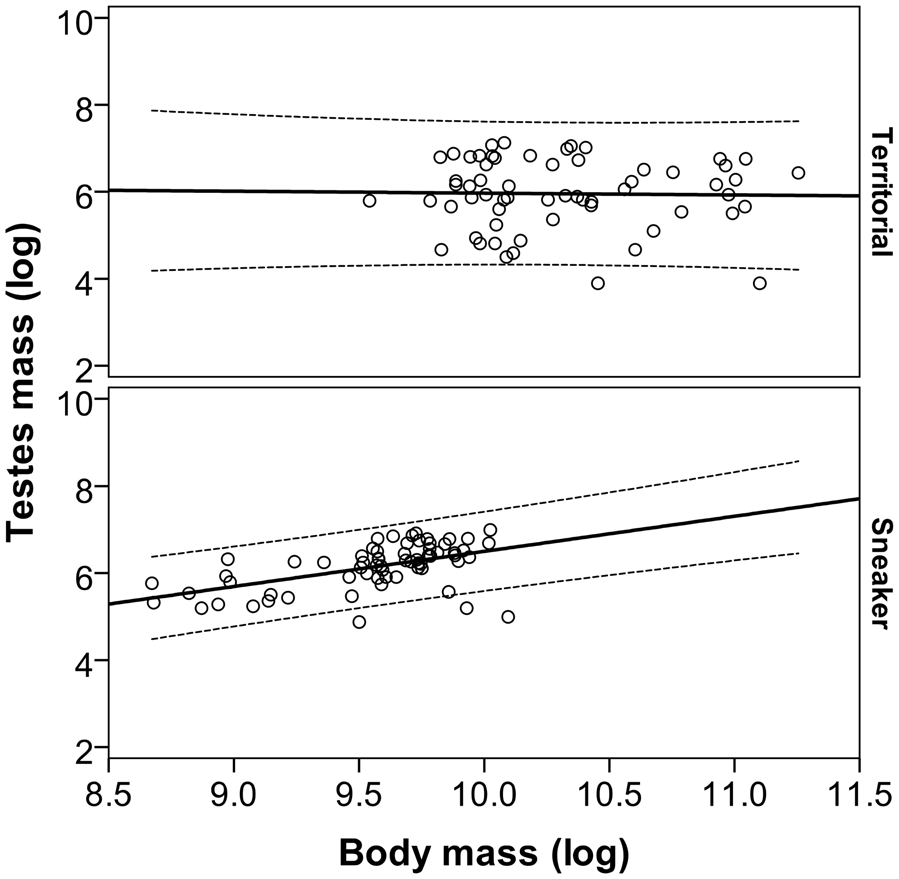
Relationship between body weight and testes weight in sneaker and territorial males of grass goby. Lines are linear regression lines and dotted lines are their 80% confidence intervals.

Gonadal investment was explored in a total of 126 grass gobies, 61 territorial males (mean body weight: 32.74±14.99 g; mean gonad weight: 0.61±0.40 g) and 65 sneakers (mean body weight: 14.99±4.34 g; mean gonad weight: 0.64±0.29 g). Territorial males presented a larger body weight (p>0.001) but did not have on average larger testes (p = 0.630). An analysis of relative testes size using ANCOVA showed a significant interaction between body weight and male category, indicating that the slopes differ for territorial and sneaker males (F_1,122_ = 7.271; p = 0.008). Correlation between body weight and testis weight was positive and significant for sneaker males (R^2^ = 0.074; R = 0.272; df = 64; p<0.001), while correlation was virtually equal to zero in territorial males (R^2^ = 0.001; R = 0.032; df = 60; p = 0.868; Figure 1).

## Discussion

### High Level of Sperm Competition in Grass Goby

Our study using microsatellite markers to estimate the paternity rates of territorial and sneaker males in the grass goby suggests a high level of sperm competition with the number of putative fathers being higher than two in all nests analyzed. The fertilization success of territorial males was high (average of 76.4%) but in all nests sneakers also contributed to the progeny. Comparing these results with paternity estimates from other fish with male parental care, the grass goby shows the highest level of cuckoldry observed, together with the fat greenling *Hexagrammos otackii* and the ocellated wrasse *Symphodus ocellatus* (reviewed in [10]). The fertilization success of sneakers in the grass goby (average of 23.56%) is higher than the values found in other fish with male nest defense, usually below 20% [16]. A considerably lower percentage of eggs sired by sneakers (9–11%) was reported in two populations of *Pomatoschistus minutus*, a species belonging to the same family [17]. Parentage genetic assignment suggested a minimum of two mothers for each nest, confirming previous observations that the grass goby presents a polygamous mating system in which territorial males monopolize more than one female [15].

In the case of the grass goby, sneakers possess larger testes relative to their body size in comparison with territorial males and increase sperm investment in order to raise their chances to fertilize eggs when they sneak into the nests [4], [15], which has also been observed in other gobies [18]. The strategy of sneakers clearly works, as our study shows that sneakers can contribute to up to 40% of the progeny. However, our data also show that the fertilization success of territorial and sneaker males is not equal, with the former group siring approximately 75% of the progeny at a given nest. In this species, males show an ontogenetic change in mating tactic, behaving as sneakers when small and young, and changing into territorial males when larger and older [15]. Moreover, it has been demonstrated that sneaker males can change tactic according to social context [19]. Therefore, it appears advantageous for a male to adopt an opportunistic tactic when smaller/younger despite lower fertilization success in comparison with territorial males, considering that sneakers can eventually become territorial males later in life, when size and/or social conditions are favorable.

### Body Size Correlates with Fertilization Success in Territorial Males

Our study shows a positive relationship between body size and fertilization success in territorial males of the grass goby. Larger territorial males both in terms of length and weight sire a larger proportion of eggs in their nest than smaller territorial males do. The lowest fertilization success was found in a territorial male with relatively small size (12.5 cm; 17.1 g), which probably represents an individual that recently changed mating tactic and became territorial. The lack of association observed between gonad size and body size suggests that males invest relatively little in sperm production once they become territorial and that the increased paternity of larger territorial males might be due to their efficiency in mate guarding in comparison with smaller territorial males.

This suggests that additional resources available after tactic change are invested in growth rather than in sperm production, as higher fertilization success might be achieved through mate and nest guarding rather than producing additional sperm. A higher investment in nest guarding as a response to sperm competition instead of adjusting sperm expenditure has been observed in many taxa [7], [20]–[23]. For instance, when given additional energy, sperm-limited males of the blue-headed wrasse increased aggression toward sneaker males instead of sperm production [24]. Alonzo and Warner [7] hypothesized that the expected fitness benefits of male tactics representing alternatives to sperm allocation should change with male size. Because size often determines the winner of aggressive interactions, the efficiency of mate guarding should increase with the relative size of the male. While there is ample evidence that this occurs across male mating tactics [25], evidence that this relationship holds also within territorial males is less ample. Our combined observations on the pattern of variation in gonadal investment and paternity, in relation to increasing body size, support this hypothesis. Large territorial males might be more effective in defending the nest than smaller territorial males. By guarding the nest better, large territorial males could chase sneakers away and decrease the number of sneaked copulations in comparison with smaller territorial males, which would explain the influence of body size on fertilization success of territorial males in the grass goby. Alternatively, the increased paternity of larger territorial males might be due to a lower intrusion rate of sneakers because a large difference in body size between territorial and sneaker males is a strong deterrent from nest intrusions.

## Materials and Methods

### Sampling Data

Permissions for fish collection were obtained from the Regione Veneto (protocols 407590/48.17, 1413/48.17, 2892/48.17, 1085/48.17). Fish manipulation and maintenance followed the guidelines of the Veterinary Service of the local “Azienda Socio Sanitaria n°14” of Chioggia (protocol 353/V). The Ethical Committee of the University of Padova (CEASA) was not established until 2006 - and our study was completed in 2004. According to Italian Law DL116/92 and European Council Directive 86/609/EEC, this study did not require authorization from the Ministry of Health because no experiments were conducted on the animals and animal manipulation was limited to marking (removal of small finclip) and sacrificing fish using the least painful method commonly accepted in modern practice as humane. Accordingly, in order to minimize the suffering of the animals used in the study, fish were brought to the laboratory in oxygenated seawater, deeply anaesthetized with MS-222 (3-aminobenzoic acid ethyl ester) dissolved in the water and dissected after severing of the spinal cord. When it was possible to avoid sacrifice, finclips were taken from anaesthetised individuals that were immediately released back to the water.

Parentage analysis was conducted in a total of 688 eggs from 8 different nests sampled during the 2003 (5–22 May) and 2004 (1 April–8 June) breeding season in the Chioggia area (45°13′09″N; 12°16′45″E) in the southern part of the Venetian lagoon (Italy; Table 1). All fish found in a given nest were taken and kept alive in seawater tanks until arrival in the laboratory (Hydrobiological Station Chioggia). Nests were screened for egg clutches, which were stored in absolute ethanol for molecular analysis. In the laboratory, all fish were anaesthesized, measured with a ruler (total length in mm), weighed (total weight in mg) and sexed based on the sexually dimorphic genital papilla [13]. After measurements were taken these fish were released where they had been caught. All nests included one territorial male, identified on the basis of length (minimum length of 12.5 cm) and the characteristics of sperm trail as described in Mazzoldi et al. [15]. Four of the nests included one small sneaker male. Four nests also included one or two females.

Since the individuals used in the genetic study were released back into the water after sampling, the association between gonad (testes) weight and body weight was tested using a sample of 126 grass gobies collected during the period 1995–1996 in the same area, including 61 territorial males (13.0–22.6 cm TL) and 65 sneakers (<12.0 cm TL). Body and gonad weight were measured for each individual to the nearest mg.

Additionally, a total of 143 adults were collected in the period 2002–2004 at four different sites of the Venice lagoon (Ca’ Zane, Lago dei Teneri, Chioggia and Lido) to be used as reference sample to test the power of the microsatellite loci for parentage analysis.

### Microsatellite and Parentage Analysis

Genomic DNA was extracted using two different protocols. Genomic DNA from adults was extracted from fin clips using the DNAeasy Blood and Tissue Extraction Kit (QIAGEN). DNA from eggs was extracted using the Gloor and Engels [26] fast protocol. All individuals were genotyped at four microsatellite loci [27], following the PCR conditions reported in the original manuscript. Individual genotyping was performed using Genotyper v. 3.7 (Applied Biosystems).

Paternity was assessed using GERUD v.2.0 [28]. All 688 sampled eggs were assigned to the candidate parents using the genotypes of territorial males, females and sneakers found in the sampled nests. When paternity was ambiguous, the simulation procedure implemented in Cervus v.2.0 [29] was used to infer the most likely parent. The minimum number of mothers contributing to each nest was also determined using GERUD. Prior to the analysis, the power of the markers for parentage assignment was estimated using the commonly used expected exclusion probabilities approach. The expected exclusion probabilities for each locus and for all loci combined were calculated according to the equations in Dodds et al. [30], using the allele frequencies observed in the reference sample of 143 adults of the Venice Lagoon.

A Monte Carlo simulation using PopTools (http://www.poptools.org) was performed in order to assess the probability of error associated with the paternity method used. At each nest, all the genotypes of the progeny were compared with the genotype of a simulated individual, randomly created using the allelic frequencies observed in the reference sample of 143 adults of the Venice Lagoon. The simulation was replicated 10,000 times and the percentage of eggs matching the simulated individual at all loci was recorded. This percentage represents the probability of a random match, i.e. the probability that an unrelated individual is not excluded by the parentage analysis.

### Statistic Analysis

A logistic regression with binomial distribution and logit link function was used to test the association between fertilization success of territorial males (number of eggs sired by territorial male over total number of eggs) and the following variables: body weight and number of sneakers and females present at the nest during sampling. Weight was used as a proxy for body size and showed a highly significant correlation with length (r = 0.98; p<0.001). A model was fit containing fertilization success of territorial males as dependent variable and predictors as covariates. Non-significant predictors were then removed in a step-wise procedure, starting from the least significant one, until only significant predictors remained in the model. The deviance increase of the generalized linear model resulting from the removal of each predictor was tested against a chi-square distribution. We also tested for a possible correlation between body weight and gonad weight using analysis of covariance (ANCOVA). Gonad weight was used as dependent variable, body weight as covariate and male category (territorial or sneaker) as factor. For allometric reasons, body weight and gonad weight were log-transformed. All statistical analyses were performed in STATISTICA v.6.0 (Statsoft Inc) and SPSS v.18.0 (SPSS Inc).
